# Exploring Personality Traits Associated with Reproductive Success in Captive Female Alpine Musk Deer (*Moschus chrysogaster*)

**DOI:** 10.3390/ani16162476

**Published:** 2026-08-10

**Authors:** Xiaoping Lu, Ni Qin, Taifu Sun, Xueyi Zhou, Xiuxiang Meng

**Affiliations:** School of Environment and Ecology, Renmin University of China, Beijing 100872, China; luxiaoping@ruc.edu.cn (X.L.);

**Keywords:** Alpine musk deer (*Moschus chrysogaster*), captive, animal personality, reproductive success, fawn mortality

## Abstract

Animal personalities, reflecting consistent individual behavioral differences, are increasingly recognized as key factors influencing fitness. However, their role in the reproductive success of endangered ungulates remains poorly understood. In this study, 17 personality traits, including “Clumsy”, “Dominant”, “Vigilant”, “Affiliative” and so on, were assessed in 123 adult female captive Alpine musk deer (*Moschus chrysogaster*) using a scale-based personality rating method. We explored the relationships between these personality traits and reproductive fitness indicators, including litter size, calving success, and fawn mortality. Our exploratory analyses revealed that “Clumsy” scores were significantly higher in females that experienced calving failure (miscarriage or dystocia) compared to those that successfully gave birth (*p* = 0.013, Cohen’s d = 0.88), indicating a large effect size. However, no traits were correlated with litter size. For fawn mortality, although initial correlation analyses suggested associations with “Vigilant” and “Affiliative”, these were not supported when a Poisson regression model—appropriate for count data—was applied with litter size as an offset (model *p* = 0.092; all predictors *p* > 0.05). These findings suggest that the previously observed associations between maternal personality and fawn mortality may have been influenced by the use of an inappropriate statistical model. Overall, our study highlights the importance of using distribution-appropriate models for count-based fitness data and provides exploratory, hypothesis-generating evidence that, while “Clumsy” may be robustly associated with calving success (large effect size), personality–fitness relationships in this species require further validation with larger samples and across different captive populations.

## Simple Summary

Individuals of the same species often differ in their consistent behavioral tendencies, known as animal personality, which can influence survival and reproduction. In this study, we assessed 17 personality traits in 123 captive female Alpine musk deer using a keeper-rating method and examined whether these traits were linked to litter size, calving success, and fawn survival. We found that females with higher scores for “Clumsy” experienced more calving difficulties. However, initial associations between maternal personality traits and fawn mortality were not supported when analyzed using a statistical model better suited for this type of data. These findings highlight the importance of selecting appropriate statistical methods for analyzing count-based fitness data and suggest that larger, independently validated studies are needed to determine whether personality traits influence reproductive outcomes in this endangered species.

## 1. Introduction

Animal personality, defined as the underlying individual behavioral tendencies that are consistent across time and contexts [1,2,3], has been documented across multiple taxa and research disciplines [4,5,6], with important ecological and evolutionary implications [7,8]. An individual’s personality traits can significantly affect their fitness [9,10]. Indeed, numerous studies have demonstrated that animal personality influences mating success [11,12], reproductive success [9,13,14], offspring weight [15,16], and survival rate [17,18,19] to varying degrees. These investigations have covered a broad range of taxa, including fish, birds, reptiles, and primates, indicating that the association between personality and fitness is widespread across the animal kingdom. However, research on personality in small ungulates, particularly endangered species such as Alpine musk deer (*Moschus chrysogaster*), remains scarce. Current studies on personality in captive musk deer have primarily focused on personality identification [20] or physiological correlates [21], but have not explicitly examined their effects on reproductive fitness.

The study of personality in captive animals presents a unique opportunity for understanding the interplay between behavioral phenotypes and fitness. Captive environments fundamentally differ from natural habitats in multiple dimensions—spatial restriction, predictable food provisioning, absence of predation, and enforced social groupings—all of which can profoundly alter the expression and adaptive value of personality traits [22]. Such environmental modifications can impose novel selective pressures that favor behavioral phenotypes distinct from those optimized in the wild. Furthermore, captivity can induce behavioral changes that are not merely transient but may reflect developmental plasticity or even heritable shifts in personality distributions across generations [23]. Understanding how personality traits respond to captive conditions and, in turn, influence reproductive success is therefore of both fundamental and applied importance, particularly for species whose long-term survival depends on successful captive breeding programs.

In addition, the relationship between fitness and personality varies considerably across species, as highlighted by the following examples. For example, in mosquitofish (*Gambusia holbrooki*), boldness was negatively correlated with its reproductive success [9], whereas the opposite pattern was observed in killifish (*Rivulus hartii*) [2]. Even among closely related species, the direction of this association between personality and fitness may vary depending on differences in ecological niches, life-history strategies, and environmental conditions. Within populations, consistent individual differences in exploration and boldness also contribute to variation in movement and resource use [24]. Such cross-species and inter-individual variability underscores the scientific value and conservation significance of conducting species-specific investigations into the personality–fitness relationship. Therefore, we suggest that exploring the relationship between personality and reproductive fitness is necessary for understanding both breeding and ex situ conservation in captive Alpine musk deer.

Musk deer (*Moschus* spp.) are rare animals that have been listed as endangered by the International Union for Conservation of Nature (IUCN) and in Appendix II of the Convention on International Trade in Endangered Species of Wild Fauna and Flora (CITES) [25,26]. However, ongoing challenges such as high abortion rates and low offspring survival have been observed in conservation programs, resulting in declining reproduction and slow population growth [27,28]. Existing studies on the fitness of musk deer have mainly focused on parental care behaviors, mating, captive environment, and feeding regimes [29,30]. However, few studies to date have explored the reproductive fitness of female musk deer from the perspective of animal personality.

Musk deer possess a suite of life-history and behavioral characteristics that make them particularly amenable to personality research. As solitary, territorial, and highly cryptic forest-dwelling ungulates, they rely heavily on behavioral strategies—such as vigilance, avoidance, and olfactory communication—to navigate ecological challenges including predation risk and resource competition [23]. Their natural social structure is minimal, with adult individuals maintaining exclusive home ranges and interacting primarily during the brief breeding season. This inherent solitude contrasts sharply with the high-density group housing typical of captive breeding facilities, where individuals are forced into frequent and often agonistic social encounters. Such a dramatic transition from a solitary to a social lifestyle likely imposes substantial behavioral and physiological stress, potentially exacerbating the expression of certain personality traits that may be maladaptive under these novel social conditions.

Animal personality, as a continuum throughout the entire life history, reflects stable behavioral differences among individuals. It offers a novel perspective for studying fitness-related issues such as parturition and survival in musk deer [31]. The evolutionary maintenance of animal personality has long been a central question in behavioral ecology. Suboptimal behavior theory [4] posits that individuals do not always exhibit optimal behaviors at all life-history stages, and that behavioral trade-offs often carry adaptive significance for the overall life history. Life-history trade-off theory further suggests that the maintenance of personality traits may be related to resource allocation conflicts among different life-history stages, where fitness benefits gained in one stage may come at the cost of reduced performance in another [32,33]. The captive environment, with its altered selective regime, offers a unique arena in which to examine these evolutionary dynamics, as traits that are neutral or beneficial in the wild may become costly under artificial conditions, and vice versa. Collectively, these theoretical frameworks provide important support for understanding the relationship between personality and reproductive fitness in captive environments.

In this study, we explored the relationship between female musk deer personality and reproductive fitness (including litter size, calving success, and offspring survival), aiming to identify personality traits (or suites of traits) that can serve as reference indicators for reproductive fitness [9,12]. Given the exploratory nature of this research and the absence of pre-registered hypotheses, we adopted a two-stage analytical approach: first screening all 17 traits for associations with reproductive outcomes, and then subjecting candidate traits to regression models appropriate for the data structure. We emphasize that our findings should be interpreted as hypothesis-generating and require independent validation.

## 2. Material and Methods

### 2.1. Research Area and Subjects

Gansu Province is one of the main distribution areas of Alpine musk deer in China. This study was conducted at the Breeding Center of Alpine musk deer (BCAMD), located within the Xinglongshan National Nature Reserve, western China (35°38′–35°58′ N, 103°50′–104°10′ E), at an elevation of 2000–2100 m above sea level, with annual rainfall of 480–622 mm and mean temperature ranging from 2.5 to 6.4 °C [25,34] over the period from June 2016 to September 2017. The facility covers 30 ha and consists of over 100 enclosures. Seven enclosures are arranged in a row, separated by brick walls and net fencing. Each enclosure is about 100 m^2^ in area and includes shaded areas such as pergola and small connecting cells linked by wooden gates. In this study, personalities were quantified using the personality ratings method on 123 adult females, all identified by ear tags (H-Bio, Shanghai, China). All individuals were housed in enclosures measuring 10 m × 10 m, and fodder, water and leaves were provided daily at 06:00–07:00 and 17:00–18:00. The only other anthropogenic interference was that the keepers cleaned the enclosure.

### 2.2. Personality Quantification and Data Collection

Current personality quantification methods include subjective personality ratings, behavioral coding and experimentation [35,36]. As all behavioral tests, such as the novel object test or flight-initiation distance test, were prohibited by BCAMD to avoid potential negative influences on reproduction and musk production in musk deer, the personality rating method was used to quantify the personalities of the animals. This approach is extensively used to quantify the personalities of African elephant (*Loxodonta africanna*) [37], fallow deer (*Dama dama*) [38] and Alpine musk deer (*Moschus chrysogaster*) [39], as it allows collection of a set of long-term representative data in a short time. Therefore, we used a scale-based personality rating method that collects data from the keepers instead of applying direct interference to the musk deer [38,40]. A total of 10 keepers, each with over two years of breeding experience, independently scored the musk deer’s personalities on a given ruler (specifically, a 7 cm linear visual analog scale with a minimum increment of 1 mm was used for scoring). The 7 cm scale was selected following established protocols in animal personality research [36,38], as it provides sufficient sensitivity for detecting individual differences while maintaining rater consistency.

In this study, 17 personality traits were assessed, each with a clear definition and scoring criteria (Table 1), with scores ranging from 1 (rarely occurs) to 7 (frequently occurs). For example, “Reckless” refers to the tendency to accidentally injure oneself or fawns through running and jumping; a score of 1 indicates that the individual rarely causes such accidental injuries, while a score of 6 indicates multiple accidental injuries caused by galloping, i.e., being more “Reckless”. To ensure reliability, we calculated the intra-class correlation coefficient (ICC) among the 10 raters for each personality trait; all ICC values exceeded 0.70, indicating good inter-observer agreement, and the mean score across raters for each individual was used in all subsequent analyses.

Disease-free individuals were selected based on fur gloss and condition, mental state, and injury status. All keepers then received centralized training to clarify the specific definitions of 17 personality traits and scoring standards prior to scoring. No exchange of views was allowed during the scoring process. Parturition data were collected from May–July 2017, and fawn mortality was defined as death occurring before weaning (within 90 days postpartum).

### 2.3. Data Analysis

Given that we assessed 17 personality traits without a pre-specified set of hypotheses regarding which traits would be associated with reproductive outcomes, we adopted a two-stage analytical approach. In the first stage, all 17 traits were screened for associations with reproductive outcomes using Spearman correlation (for fawn mortality and litter size) and independent-samples *t*-tests (for calving success), with these analyses treated as exploratory. In the second stage, traits showing significant or suggestive associations in the exploratory screening—specifically, Vigilant, Affiliative, and Dominant for fawn mortality—were entered together into a Poisson regression model to assess their independent effects while accounting for litter size as an offset.

We used correlation tests and regression models to assess relationships among variables (including the relationship between personality score and litter size, calving success, and mortality), and used the Shapiro–Wilk test to check for normality. When original variables were continuous and normally distributed, we performed correlation tests using scatter plots combined with Pearson’s product-moment correlation analysis. Otherwise, Spearman’s rank correlation coefficient was used for non-parametric analysis. This two-stage design was necessary given the sample size constraints (*n* = 105 females with known litter size) and the need to avoid overfitting.

For the Poisson regression, we used a generalized linear model with a Poisson distribution and a log-link function, including the natural logarithm of litter size as an offset variable to account for variation in litter size across females. Multicollinearity among predictors was checked using variance inflation factors (VIF), with values below 10 considered acceptable. Effect sizes for the *t*-test were calculated as Cohen’s d. Model fit was assessed using the deviance/df ratio, with values < 1.5 indicating no overdispersion. Results from the exploratory correlation analyses should be interpreted as hypothesis-generating rather than confirmatory; no correction for multiple testing was applied to the exploratory correlations, consistent with their screening purpose. However, we acknowledge the increased risk of Type I error and have interpreted the findings cautiously throughout. All analyses were performed using SPSS 20 (IBM Corp., Armonk, NY, USA), and results are presented as mean ± SD for descriptive statistics and as B ± SE for regression estimates, and were considered significant at *p* < 0.05.

### 2.4. Ethical Statement

This study was approved by the Institutional Animal Care and Use Committee of Renmin University of China and adhered to the regulations of the Breeding Center of Alpine musk deer (BCAMD). All procedures relied exclusively on non-invasive behavioral observations and routine management records, and no invasive manipulations were conducted. All animal procedures complied with the Wild Animals Protection Law of the People’s Republic of China and followed international ethical standards for animal research.

## 3. Results

### 3.1. Personality Correlates of Calving Success

Descriptive statistics for all 17 personality traits assessed in the 123 captive female Alpine musk deer are summarized in Table 2. The overall mean scores indicated moderate variability across traits, with “Cautious” (4.84 ± 1.55) and “Escaped” (4.42 ± 1.37) receiving relatively higher scores, whereas “Aggressive” (3.05 ± 1.31), “Defiant” (3.13 ± 1.04) and “Reckless” (3.19 ± 1.55) received lower scores, suggesting the distribution of behavioral phenotypes in this captive population.

Spearman’s rank correlation tests revealed that none of the 17 personality traits were significantly correlated with litter size (all *p* > 0.05; *rs* ranging from −0.102 to 0.118), indicating that maternal personality did not influence the number of offspring produced per parturition. These correlations were examined as part of an exploratory screening for traits potentially associated with reproductive outcomes and should be interpreted with appropriate caution.

**Figure 1:**
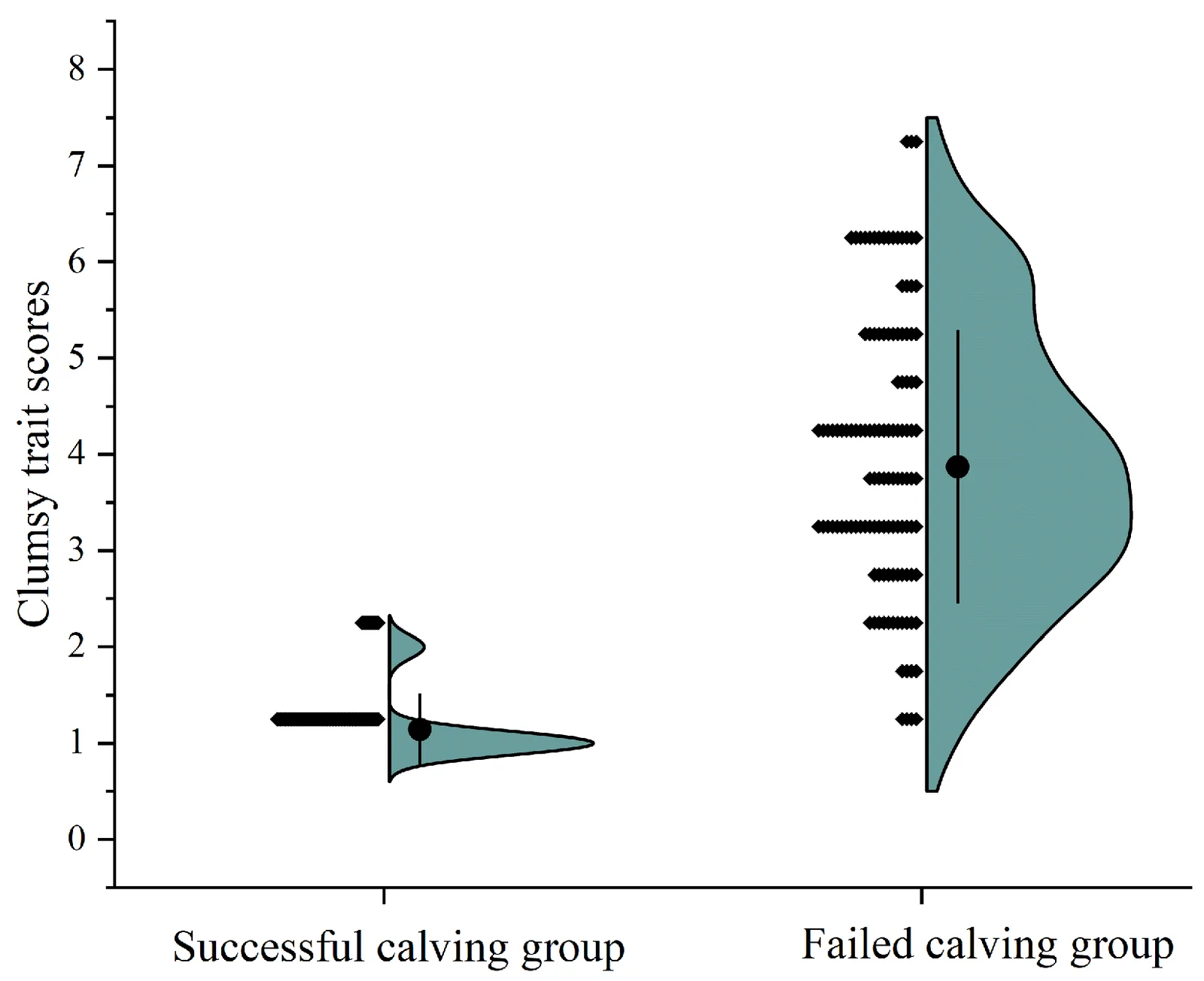
Violin plot showing the distribution of “Clumsy” personality scores across two reproductive outcome groups: females with successful calving and females experiencing reproductive failure (miscarriage or dystocia). The short horizontal lines on the left represent individual data points; the right violin shape indicates data density. Central dots denote group mean values.

However, “Clumsy” was significantly associated with calving outcomes. Females that successfully gave birth (*n* = 105) had a significantly lower “Clumsy” score (3.78 ± 0.14) compared to those that experienced miscarriage or dystocia (*n* = 18; 4.44 ± 0.89; independent-samples *t*-test: t = −2.615, df = 34.897, *p* = 0.013; Table 3). The effect size was large (Cohen’s d = 0.88); however, given the small sample size of the failed-calving group (*n* = 18), this estimate should be interpreted with caution. The calving success rate was 85.4% for individuals with “Clumsy” scores below the median (<4.0, *n* = 63), whereas it decreased to 80.0% for those with scores above 4.5 (*n* = 55), further supporting the negative impact of this trait on reproductive success. The distribution of “Clumsy” scores across the two calving outcome groups is visualized in Figure 1.

**Figure 2:**
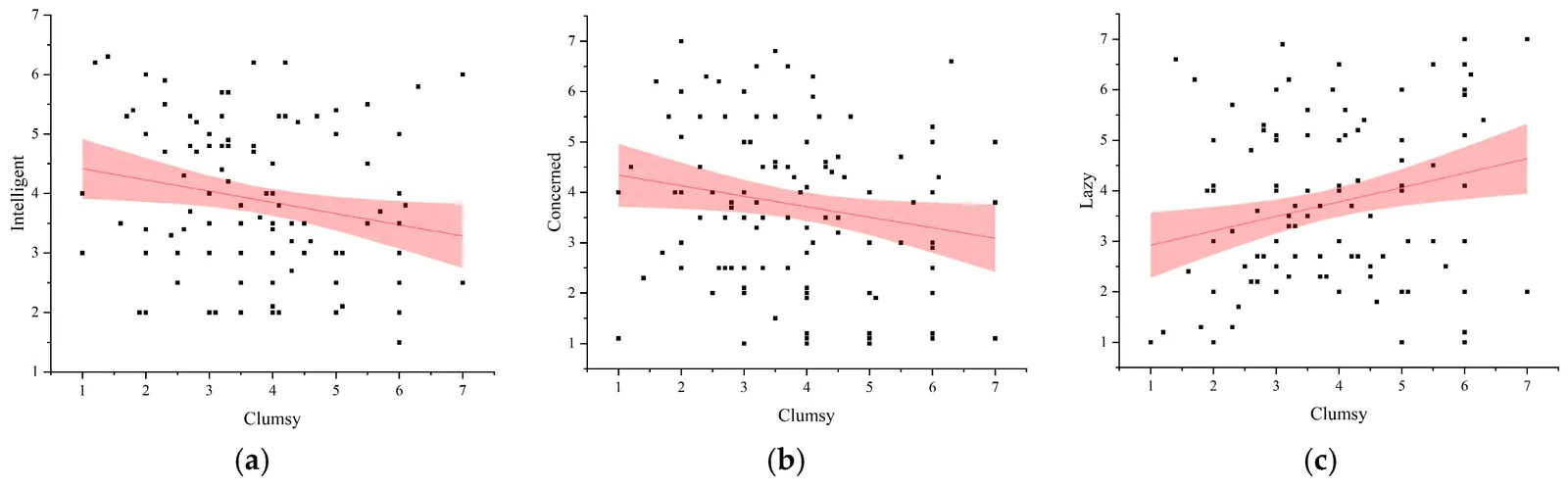
Relationships between “Clumsy” and (**a**) “Intelligent”, (**b**) “Affiliative”, (**c**) “Lazy” in captive female Alpine musk deer.

To further characterize the behavioral profile underlying “Clumsy”, we examined its correlations with other personality traits (Figure 2). “Clumsy” was significantly negatively correlated with “Intelligent” (r = −0.211, *p* = 0.019; Figure 2a), indicating that clumsier individuals were less perceptive and less exploratory. It was also negatively correlated with “Affiliative” (r = −0.188, *p* = 0.037; Figure 2b), suggesting social withdrawal and reduced peer contact. Conversely, “Clumsy” was positively correlated with “Lazy” (r = 0.246, *p* = 0.006; Figure 2c), confirming that these individuals tended to be inactive and sedentary. Collectively, these correlational patterns suggest that “Clumsy” represents a composite behavioral syndrome characterized by low exploration, low sociability, and high inactivity, which may collectively predispose females to calving failure under captive conditions.

### 3.2. Maternal Personality Traits Associated with Fawn Mortality

Among the 105 females that successfully gave birth, we analyzed the relationship between maternal personality traits and pre-weaning fawn mortality (within 90 days postpartum). The mean mortality rate per female was 0.68 ± 0.73. Spearman’s rank correlation tests revealed that two personality traits were significantly positively correlated with fawn mortality: “Vigilant” (r = 0.235, *p* = 0.008) and “Affiliative” (r = 0.233, *p* = 0.008). “Dominant” showed a positive but non-significant trend (r = 0.155, *p* = 0.057). Based on the exploratory correlations, we proceeded to hypothesis-driven regression analyses for the traits that showed significant (Vigilant, Affiliative) or suggestive (Dominant) associations with fawn mortality.

Because fawn mortality was recorded as count data (0, 1, or 2 deaths per female) and required consideration of litter size as an exposure variable, we fitted a generalized linear model with a Poisson distribution and a log-link function, using the natural logarithm of litter size as an offset. The overall model was not statistically significant (likelihood ratio χ^2^ = 6.448, df = 3, *p* = 0.092). None of the three personality traits examined—Vigilant, Affiliative, or Dominant—emerged as significant predictors of fawn mortality (all *p* > 0.05; Table 4). Variance inflation factors (VIF) for all predictors were < 1.15 (range: 1.050–1.143), indicating no multicollinearity. The model showed no evidence of overdispersion (Deviance/df = 0.805; Pearson χ^2^/df = 0.637), supporting the use of Poisson regression. The incidence rate ratios (IRR) for Vigilant, Affiliative, and Dominant were 1.137 (95% CI: 0.938–1.377), 1.102 (95% CI: 0.940–1.293), and 1.067 (95% CI: 0.929–1.225), respectively, with all 95% confidence intervals crossing 1, further confirming the lack of statistically significant effects.

**Figure 3:**
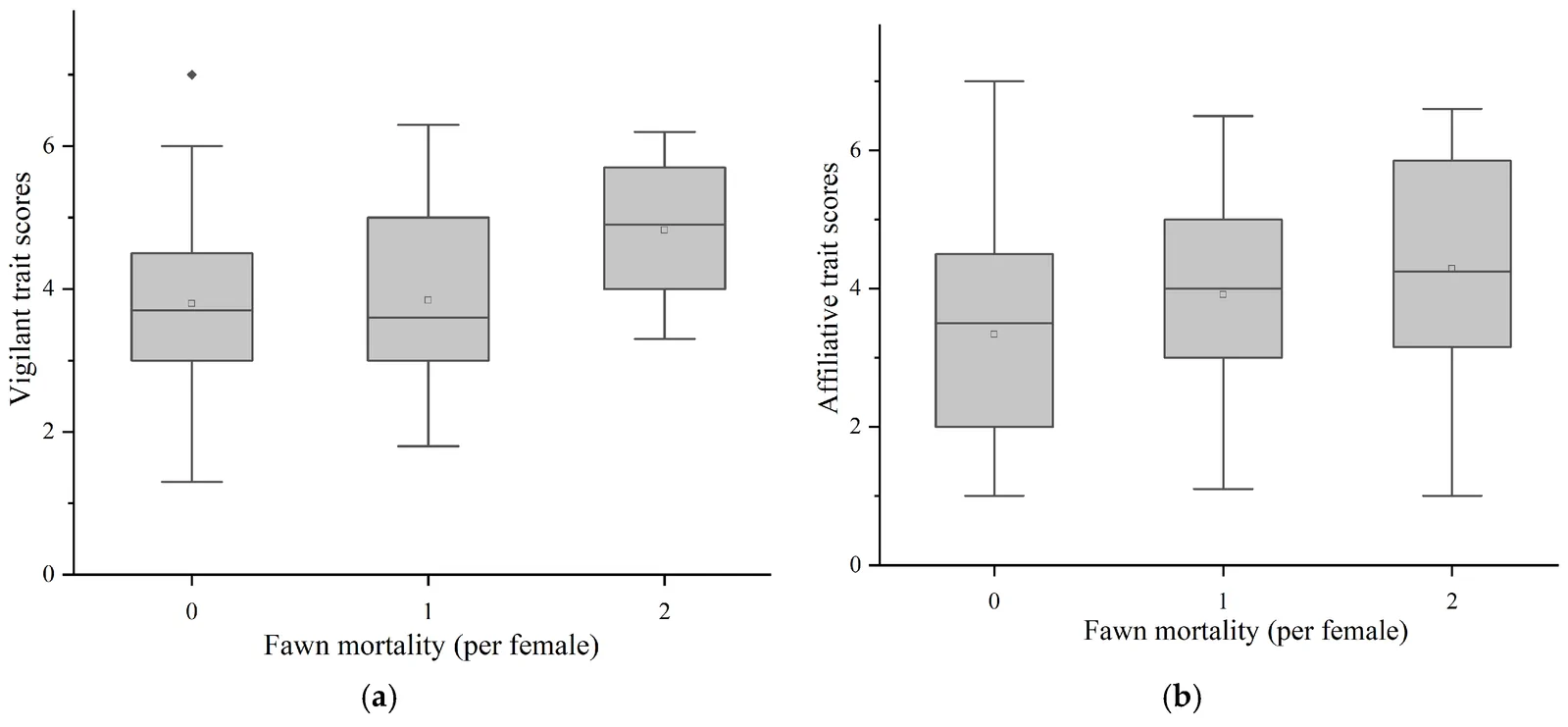
Boxplots illustrating the distribution of maternal (**a**) “Vigilant” and (**b**) “Affiliative” personality scores stratified by pre-weaning fawn mortality count per female (0, 1, 2 dead fawns; *n* = 105 successfully calving females). Box midlines represent median scores, and hollow squares indicate group mean values.

These results indicate that the previously observed significant correlations between maternal personality traits and fawn mortality in the exploratory screening were not supported when analyzed using a statistical model appropriate for count data and accounting for litter size. The distributions of “Vigilant” and “Affiliative” scores across different fawn mortality counts are presented in Figure 3 for descriptive purposes.

## 4. Discussion

This study provides exploratory, hypothesis-generating evidence linking personality traits to reproductive fitness in captive female Alpine musk deer, a relationship that has remained largely unexplored in small, endangered ungulates. Our initial exploratory screening suggested associations between specific personality traits and reduced reproductive outcomes. However, after applying statistical models appropriate for the data structure, only the association between “Clumsy” and calving success was supported, whereas the hypothesized links between “Vigilant”/”Affiliative” and fawn mortality were not confirmed. These results not only corroborate the general premise that personality has fitness consequences but also highlight the critical importance of using distribution-appropriate statistical methods and the need for cautious interpretation of exploratory findings in behavioral ecology. We emphasize that the patterns reported here should be treated as preliminary and hypothesis-generating, pending independent validation.

### 4.1. Clumsy and Calving Success

Individuals with high “Clumsy” were more susceptible to abortion and dystocia, resulting in lower reproductive success overall. Our statistical comparisons revealed that females that experienced calving failure had significantly higher “Clumsy” scores (4.44 ± 0.89) than those that successfully gave birth (3.78 ± 1.46; independent-samples *t*-test: t = −2.615, df = 34.897, *p* = 0.013; Table 3), supporting a robust bivariate association between this trait and parturition success. The effect size was large (Cohen’s d = 0.88); however, given the small sample size of the failed-calving group (*n* = 18), this estimate should be interpreted with caution, as the confidence interval around the effect size is likely to be wide. The “Clumsy” trait partly represents stereotypic behaviors, indicating that stereotypic behaviors have a negative impact on reproductive success, especially behaviors such as wall-jumping, platform-standing and to-and-fro walking. This is consistent with the results of Meng et al. [25] and further verifies the utility of stereotypic behaviors as indicators of stress and reduced welfare [12,41,42]. However, it should be noted that stereotypic behaviors can have different impacts across life-history stages and may sometimes be beneficial [43]; Low-intensity stereotypies may serve as a coping mechanism [44], and species differ in their responses to captivity [22]. Studies on captive musk deer have reported similar patterns [45,46], and social stress can further influence welfare [47].

In addition, “Clumsy” also partially reflects an individual’s activity level, including environmental exploration and social interactions. The significant correlational patterns we observed— that “Clumsy” was negatively correlated with “Intelligent” (r = −0.211, *p* = 0.019) and “Affiliative” (r = −0.188, *p* = 0.037), but positively correlated with “Lazy” (r = 0.246, *p* = 0.006; Figure 2)—provide empirical evidence for the existence of a behavioral syndrome. Specifically, individuals that are more active, more exploratory, and that initiate more contact with peers tend to have better reproductive performance, suggesting a stable combination of personalities—i.e., behavioral syndromes [4]. Less “Clumsy” females tended to be more “Intelligent” (environmentally perceptive) and more “Affiliative” (socially engaged), implying a proactive coping style that facilitates better adaptation to the captive environment. Conversely, high “Clumsy” individuals showed a reactive or passive profile, exhibiting low exploration and social withdrawal. This dichotomy aligns with the proactive-reactive coping style axis observed in other vertebrates [48]. In the context of captive breeding, the proactive phenotype appears to confer a reproductive advantage, likely because these individuals are better at exploiting available resources and navigating social dynamics without incurring the physiological costs associated with chronic stress. The descriptive data in Table 2 further indicate that our captive population exhibited substantial inter-individual variation in all 17 traits providing a solid foundation for detecting these personality–fitness relationships, but whether these traits have longer-term consistency and heritability still requires further investigations.

More broadly, the inactivity and stereotypic behaviors associated with by “Clumsy” may arise from discrepancies between artificial captivity and natural habitats, such as spatial restriction, fixed-time feeding, and rationed food. Stereotypic behavior is considered an adaptation to captive stress and is a unique feature of captive wild animals [23,29]. Not all individuals exhibit stereotypies, and therefore we speculate that acquired environmental factors and experience (e.g., artificial hand-rearing) impose differential selection pressures, leading to diverse personalities or behavioral syndromes.

### 4.2. Maternal Personality Traits and Pre-Weaning Fawn Mortality

Contrary to our initial expectations based on exploratory correlation analyses, the Poisson regression analysis—which is statistically appropriate for count data such as fawn mortality and accounts for litter size as an exposure variable—did not support a significant association between any of the three examined personality traits (Vigilant, Affiliative, or Dominant) and fawn mortality. The overall model was not significant (likelihood ratio χ^2^ = 6.448, df = 3, *p* = 0.092), and none of the individual predictors reached statistical significance (all *p* > 0.05; Table 4). The incidence rate ratios for Vigilant, Affiliative, and Dominant were 1.137 (95% CI: 0.938–1.377), 1.102 (95% CI: 0.940–1.293), and 1.067 (95% CI: 0.929–1.225), respectively, with all 95% confidence intervals crossing 1.

These results indicate that the previously observed significant correlations between “Vigilant”/”Affiliative” and fawn mortality in the exploratory screening were not supported when analyzed using a model that appropriately handles the count nature of the response variable and accounts for variation in litter size. This outcome highlights the importance of selecting distribution-appropriate models for count-based fitness data and suggests that the earlier associations may have been false-positive findings attributable to the use of an inappropriate statistical framework.

Although we had hypothesized, based on life-history trade-off theory [32,33], that defensive vigilance behaviors or heightened social engagement might impose energetic costs that reduce maternal investment, these mechanistic interpretations were not directly tested in this study. In the absence of physiological measurements (e.g., glucocorticoid or androgen metabolites), metabolic rates, or direct behavioral observations of maternal care, such explanations remain speculative and require empirical testing in future work.

We therefore interpret our findings regarding fawn mortality as preliminary and hypothesis-generating, and emphasize that independent replication in larger datasets is required before any firm conclusions can be drawn regarding the relationship between maternal personality and offspring survival in this species.

### 4.3. Limitations

Several methodological considerations should be borne in mind when interpreting our findings.

First, the association between “Clumsy” and calving failure, while showing a large effect size (Cohen’s d = 0.88), was based on a small subsample of only 18 females that experienced miscarriage or dystocia. This small sample size limits the precision of the effect size estimate and precludes robust control for potential confounders such as female age, parity, and health history, which were not available in our dataset. The small sample size also increases the risk that the observed association may be influenced by outliers or unmeasured variables. Therefore, this finding should be considered hypothesis-generating and requires replication in larger, independent populations.

Second, the exploratory screening of 17 personality traits against multiple reproductive outcomes inevitably inflates the risk of Type I error. We have therefore interpreted correlations as hypothesis-generating and focused our primary inference on the regression analyses, which examined a limited set of traits selected based on the screening results. Nevertheless, even these regression findings should be considered preliminary and require validation in independent populations before firm conclusions can be drawn.

Third, our study relied exclusively on keeper-based personality ratings. While inter-rater reliability (ICC > 0.70) was satisfactory, we cannot exclude the possibility that raters’ knowledge of individuals’ reproductive histories may have influenced their assessments, nor can we confirm the temporal stability of the rated traits due to the absence of longitudinal validation. Ratings may reflect behavioral frequency or transient states rather than stable personality dimensions. Future studies should incorporate repeated behavioral observations and, where feasible, experimental assays to complement rating-based measures.

Fourth, several potential confounders—such as female age, parity, enclosure conditions, and health status—were not included in our analyses due to data limitations. We acknowledge that these variables may influence both personality expression and reproductive outcomes, and their omission should be considered when evaluating the generalizability of our results.

Fifth, our sample was drawn from a single captive population under specific management conditions. Caution is therefore warranted when extrapolating our findings to wild populations or to other captive facilities with different husbandry practices.

Collectively, these limitations highlight the need for independent replication, longitudinal designs, and integration of physiological measures to strengthen causal inferences regarding the relationship between personality and reproductive fitness in this endangered species.

## 5. Conclusions

Animal personality provides a new perspective for endangered animal conservation [4], but research on small ungulates remains limited. In this study, we investigated captive female Alpine musk deer and explored the relationship between personality and reproductive fitness. Our findings indicate that the “Clumsy” trait—characterized by poor motor coordination, stereotypic movements, and low activity levels—was associated with a higher likelihood of calving failure (miscarriage or dystocia). This suggests that stereotypic-prone or inactive behavioral phenotypes may face greater reproductive challenges under captive conditions, although this finding requires replication in larger and independent populations.

However, contrary to our initial expectations, the hypothesized associations between maternal personality traits (“Vigilant”, “Affiliative”, and “Dominant”) and pre-weaning fawn mortality were not supported when analyzed using a statistical model appropriate for count data that accounted for litter size. This outcome highlights a key methodological lesson: the choice of statistical framework can materially influence conclusions drawn from count-based fitness data, and exploratory correlations should be interpreted with appropriate caution.

These findings should be regarded as exploratory and hypothesis-generating, rather than confirmatory, given the modest sample size, the single-population design, and the absence of physiological and longitudinal data. For the development of ex situ conservation and breeding programs, our results offer preliminary insights rather than definitive management recommendations. While the Clumsy–calving association may offer a potential behavioral indicator for identifying females at elevated reproductive risk, we emphasize that these results require validation across independent populations and varied management conditions before any practical applications can be recommended.

Future research integrating physiological measures (e.g., glucocorticoid metabolites), longitudinal behavioral assessments, and controlled experimental designs will be essential to establish causal relationships and to determine whether personality-based screening can meaningfully improve reproductive outcomes in captive breeding programs. Overall, this study contributes to the growing body of exploratory research on captive animal personality and provides a methodological caution for future studies investigating personality–fitness relationships in endangered species.

## Figures and Tables

**Table 1:** Initial personality scale of captive Alpine musk deer (17 personality items).

Number	Item	Definition
1	Reckless	Running and leaping resulting in accidental injury to fawns or themselves.
2	Active	Spending more time wandering or sniffing than reposing.
3	Aggressive	Displaying threat behaviors towards people or attacks on peers.
4	Dominant	Displacing others, feeding in priority and depriving others of food during feeding.
5	Cautious	Sensitive, and frequently standing gazing with binaural rotation to avoid potential injury.
6	Irritable	Prone to threats and attacks when areas are being swept and food is being replaced.
7	Decisive	Proactively approaching peers and showing amicable interactions, such as mutual grooming.
8	Defiant	Behaving inconsistently with the social rank order, and actively vying for feeding priority but often incurring threats and attacks.
9	Intelligent	Quickly and accurately judging and adapting to the new environment.
10	Vigilant	Showing defensive behaviors (gaze, stamp and vigilant blowing, etc.) in social contexts.
11	Clumsy	Uncoordinated in activities such as moving, wall-jumping and platform-standing, being unable to complete the corresponding behavior properly.
12	Unsteady	Display repetitive and persistent stereotyped behaviors such as to-fro-walking, walking along a wall, etc.
13	Friendly	Seeking close contact with peers and seldom initiating hostile behaviors.
14	Anxious	Being in a restless state, showing jitteriness and open-mouth breathing, etc.
15	Lazy	Preferring to stay in enclosures, reluctant to move or move slowly, and avoid vigorous moving.
16	Affiliative	Showing scent-marking and rubbing to peers when feeding or reposing.
17	Escaped	Showing flight and retreat when faced with potential disturbances or attacks.

Footnote: The behavioral labels used in this study are defined operationally based on observable behaviors rather than psychological states. For example, “Vigilant” refers to defensive displays (gaze, stamping, alert vocalization) in social contexts, “Affiliative” refers to olfactory and physical contact behaviors (scent-marking, rubbing) during feeding or resting, and “Clumsy” refers to poor motor coordination and stereotypic movements (wall-jumping, pacing, platform-standing). While these labels may differ from standard personality constructs, they were derived from preliminary behavioral observations and keeper interviews specific to musk deer ethology.

**Table 2:** Descriptive statistics of 17 personality traits for 123 captive female Alpine musk deer. All personality scores ranged from 1 (rare occurrence) to 7 (frequent occurrence).

Personality Trait	Mean	SD
Dominant	4.03	1.79
Cautious	4.84	1.55
Reckless	3.19	1.55
Active	3.47	1.43
Aggressive	3.05	1.31
Irritable	3.48	1.46
Decisive	4.07	1.38
Defiant	3.13	1.04
Intelligent	3.87	1.26
Vigilant	3.99	1.26
Clumsy	3.87	1.40
Unsteady	3.38	1.37
Friendly	3.62	1.57
Anxious	3.44	1.19
Lazy	3.74	1.63
Affiliative	3.74	1.56
Escaped	4.42	1.37

**Table 3:** Comparison of “Clumsy” scores between successful and failed calving groups in captive female Alpine musk deer (independent-samples *t*-test assuming unequal variances).

Reproductive Outcome	*n*	Clumsy Score (Mean ± SD)	t	df	*p*-Value	Cohen’s d
Successful calving	105	3.78 ± 1.46	t = −2.615	34.897	0.013	0.88
Failed calving (abortion/dystocia)	18	4.44 ± 0.89				

**Table 4:** Generalized linear model (Poisson regression) for predictors of fawn mortality in captive female Alpine musk deer (*n* = 105, only successfully calving females included).

Variable	B	SE	Wald χ^2^	df	*p*	IRR	95% CI for IRR
(Intercept)	−1.958	0.503	15.130	1	<0.001	0.141	(0.053, 0.379)
Vigilant	0.128	0.098	1.710	1	0.191	1.137	(0.938, 1.377)
Affiliative	0.097	0.082	1.427	1	0.232	1.102	(0.940, 1.293)
Dominant	0.065	0.071	0.839	1	0.360	1.067	(0.929, 1.225)

Footnote: Offset variable = ln (LitterSize). IRR = incidence rate ratio; CI = confidence interval.

## Data Availability

The data presented in this study are available on request from the corresponding author.

## References

[1] Stamps J., Groothuis T. (2010). The development of animal personality: Relevance, concepts and perspectives. Biol. Rev..

[2] Guenther A. (2018). Life-history trade-offs: Are they linked to personality in a precocial mammal (*Cavia aperea*)?. Biol. Lett..

[3] Caspi A., Roberts B.W., Shiner R.L. (2005). Personality development: Stability and change. Annu. Rev. Psychol..

[4] Sih A., Bell A., Johnson J.C. (2004). Behavioral syndromes: An ecological and evolutionary overview. Trends Ecol. Evol..

[5] Bell A.M. (2007). Animal personalities. Nature.

[6] Nettle D. (2006). The evolution of personality variation in humans and other animals. Am. Psychol..

[7] Biro P.A., Stamps J.A. (2008). Are animal personality traits linked to life-history productivity?. Trends Ecol. Evol..

[8] Gurven M., von Rueden C., Stieglitz J., Kaplan H., Rodriguez D.E. (2014). The evolutionary fitness of personality traits in a small-scale subsistence society. Evol. Hum. Behav..

[9] Wilson A.D.M., Godin J.G.J., Ward A.J.W. (2010). Boldness and reproductive fitness correlates in the eastern mosquitofish, *Gambusia holbrooki*. Ethology.

[10] Sih A., Cote J., Evans M., Fogarty S., Pruitt J. (2012). Ecological implications of behavioural syndromes. Ecol. Lett..

[11] Ariyomo T.O., Watt P.J. (2012). The effect of variation in boldness and aggressiveness on the reproductive success of zebrafish. Anim. Behav..

[12] Korhonen H.T., Jauhiainen L., Rekila T. (2002). Effect of temperament and behavioural reactions to the presence of a human during the pre-mating period on reproductive performance in farmed mink (*Mustela vison*). Can. J. Anim. Sci..

[13] Smith B.R., Blumstein D.T. (2008). Fitness consequences of personality: A meta-analysis. Behav. Ecol..

[14] Dingemanse N.J., Both C., Drent P.J., Tinbergen J.M. (2004). Fitness consequences of avian personalities in a fluctuating environment. Proc. R. Soc. B.

[15] Stapley J., Keogh J.S. (2005). Behavioral syndromes influence mating systems: Floater pairs of a lizard have heavier offspring. Behav. Ecol..

[16] Réale D., Bianchet M.F.B. (2003). Predator-induced natural selection on temperament in bighorn ewes. Anim. Behav..

[17] Seyfartha R.M., Silkb J.B., Cheneyc D.L. (2012). Variation in personality and fitness in wild female baboons. Proc. Natl. Acad. Sci. USA.

[18] Silk J., Alberts S., Altmann J. (2003). Social bonds of female baboons enhance infant survival. Science.

[19] Armitage K.B. (1986). Individuality, social behavior, and reproductive success in yellow-bellied marmots. Ecology.

[20] Yang S., Zhang T.X., Li Y.M., Xu S.H., Zhang M.S., Hu X., Liu S.Q., Hu D.F. (2021). Identifying personality traits and their potential application to the management of captive forest musk deer (*Moschus berezovskii*). Appl. Anim. Behav. Sci..

[21] Huang W., Wang X.L., Zhou X., Wu J.Y., Lu X.P., Shakun V., Solovej I., Li Y., Zhou M., Meng X.X. (2024). Relationships between boldness and fecal steroid hormone levels in captive alpine musk deer (*Moschus chrysogaster*) in China. Appl. Anim. Behav. Sci..

[22] Mason G.J. (2010). Species differences in responses to captivity: Stress, welfare and the comparative method. Trends Ecol. Evol..

[23] Price E.O. (1984). Behavioural aspects of animal domestication. Q. Rev. Biol..

[24] Fraser D.F., Gilliam J.F., Daley M.J., Le A.N., Skalski G.T. (2001). Explaining leptokurtik movement distributions: Intrapopulation variation in boldness and exploration. Am. Nat..

[25] Meng X.X., Feng J., Yun M., Wang B., Cody N. (2011). Relationships between musk extraction, social rank and tail-rubbing in male Alpine musk deer, *Moschus sifanicus*. Biologia.

[26] Meng X.X., Liu D., Feng J., Meng Z. (2012). Asian Medicine: Exploitation of Wildlife. Science.

[27] Geng S., Ma S. (2000). Decline of musk deer in China and prospects for management. Environ. Conserv..

[28] Zhou Y., Meng X.X., Feng J., Yang Q.S., Feng Z., Xia L., Bartos L. (2004). Review of the distribution, status and conservation of musk deer in China. Folia Zool..

[29] Meng X.X., Yang Q.S., Feng Z., Xue H. (2007). Stereotyped behavior of captive (*Moschus sifanicus*) in Xinglong Mountain Nature Reserve. Chin. J. Ecol..

[30] Xu Z.Q., Xu H.F. (2002). The social behavior in female musk deer and its influence on offspring’s survival. Acta Theriol. Sin..

[31] Wilson D.S., Clark A.B., Coleman K., Dearstyne T. (1994). Shyness and boldness in humans and other animals. Trends Ecol. Evol..

[32] Wolf M., Van Doorn G.S., Leima O., Weissing F.J. (2007). Life-history trade-offs favour the evolution of animal personalities. Nature.

[33] Wolf M., Weissing F.J. (2010). An explanatory framework for adaptive personality differences. Philos. Trans. R. Soc. B.

[34] Meng X.X., Yang Q.S., Xia L., Feng Z., Jiang Y., Wang P.M. (2003). The temporal estrous patterns of female alpine musk deer in captivity. Appl. Anim. Behav. Sci..

[35] Highfill L., Hanbury D., Kristiansen R., Kuczaj S., Watson S. (2010). Rating vs. coding in animal personality research. Zoo Biol..

[36] Carter A.J., Feeney W.E., Marshall H.H., Cowlishaw G., Heinsohn R. (2013). Animal personality: What are behavioural ecologists measuring?. Biol. Rev..

[37] Freeman E.W., Schulte B.A., Brown J.L. (2010). Using behavioral observations and keeper questionnaires to assess social relationships among captive female African elephants. Zoo Biol..

[38] Bergvall U.A., Schapers A., Kjellander P., Weiss A. (2011). Personality and foraging decisions in fallow deer, Dama dama. Anim. Behav..

[39] Sun T.F., Wang J., Li Y., Zhou M., Meng X.X. (2018). The classification of personality in captive Alpine musk deer (*Moschus sifanicus*). Acta Theriol. Sin..

[40] Watters J.V., Powell D.M. (2012). Measuring animal personality for use in population management in zoos: Suggested methods and rationale. Zoo Biol..

[41] Mason G. (1992). Stereotypy and welfare in mink. J. Anim. Sci..

[42] Shrestha M.N. (1998). Animal welfare in the musk deer. Appl. Anim. Behav. Sci..

[43] Fraser A.F., Broom D.M. (1990). Farm Animal Behaviour and Welfare.

[44] Mason G., Clubb R., Latham N., Vickery S. (2007). Why and how should we use environmental enrichment to tackle stereotypy behaviour?. Appl. Anim. Behav. Sci..

[45] Han H.J., Qiao G.L., Cai Y.H., Cheng J.G., Fu W.L., Meng X.X. (2018). Stereotypic behavior of captive forest musk deer (*Moschus berezovskii*). Chin. J. Zool..

[46] Qin Y.H., Wang M.Y., Li Y., Zhou M., Bai S.L., Meng X.X. (2020). Relationships between stereotypical behavior and fecal steroid hormone in captive Alpine musk deer (*Moschus chrysogaster*). Acta Theriol. Sin..

[47] Zhou X., Lv Q.X., Qin Y.H., Yuan N.X., Li Y., Zhou M., Meng X.X. (2023). Effects of social stress on the welfare of captive male Alpine musk deer: Stereotypic behavior, fecal cortisol, and musk secretion. Appl. Anim. Behav. Sci..

[48] Koolhaas J.M., Korte S.M., De Boer S.F., Van Der Vegt B.J., Van Reenen C.G., Hopster H., De Jong I.C., Ruis M.A., Blokhuis H.J. (1999). Coping styles in animals: Current status in behavior and stress-physiology. Neurosci. Biobehav. Rev..

